# Fowler’s Solution

**Published:** 1882-11

**Authors:** E. C. Seguin


### Fowler's Solution.

By E. C. Seguin.

In this preparation, ℳj contains $\frac{1}{120}$ grain or gm. .0005 of arsenious acid.

Doses as given by authorities on Materia Medica and Therapeutics:

Stillé and Maisch, National Dispensatory (1879), p. 864, state only that the commencing dose is 5 drops (which is too much for some cases).

Stillé—Therapeutics (1874), vol. ii, p. 811. "Dose, 5 drops, generally increased to 10 or 20 drops three times a day, largely diluted and taken after meals."

Wood—Therapeutics (1880), p. 385. "Average commencing dose is 5 drops in a wine-glassful of water after meals, to be increased." Page 382, "the dose must be steadily increased until œdema or other manifestations betray its decided action." [In chorea.]

Bartholow—Materia Medica (1880), p. 124. "Dose, from 2 to 10 minims. In this disease [speaking of chorea] large doses —5 minims *ter in die*—must be given." p. 130 (!).

Rice—Posological Tables (1879), p. 45. "The dose is 3 to 10 minims and more, with caution."

Nothnagel and Rossbach—Arzneimittellehre (1878), p. 212. "Dose, from 2 to 5 drops three times a day."

Gubler—Leçons de Thérapeutique (1877), p. 100. "Dose, 5 to 20 drops per diem."

Doses as given by authorities on Clinical Medicine:

Austin Flint, Sr.—Practice of Medicine (1881), p. 811, speaking of the treatment of chorea, says: "Fowler's solution is the most eligible preparation. Commencing with 3 or 4 drops three times daily, the dose should be gradually increased until the characteristic effects are observed."

Nothing is said of the mode of administration, or of the maximal dose. I must confess to a doubt whether this eminent teacher and practitioner does use really efficient doses, for he says, also on p. 811, "of the divers remedies indicated, none can be relied upon for promptly arresting the course of the disease, and it is doubtful if any exert a special curative agency."

C. B. Radcliffe, Reynold's System of Medicine [American edition, 1879], vol. i, p. 712, article Chorea, correctly and fully describes the progressive and fearless dosage of this remedy, increased from 5 drops after each meal to saturation of the system. If gastric derangement occurs, he suspends treatment for a while and then goes on. He quotes Dr. Begbie as giving it in the same way, and he has never known it to fail in thirty years.

Meigs and Pepper, Diseases of Children (1870), p. 564, quote Radcliffe, and add, "Fowler's solution given in ordinary doses and immediately after eating, and steadily persisted in until some evidence of its constitutional effects are observed."

If these writers correctly express their meaning, simply "persisting" in ordinary doses (5 to 10 drops) is the proper method. But it is by just such a method that chorea may be perpetuated and a belief in the non-efficiency of drugs engendered in the physician's mind.

J. Lewis Smith, Diseases of Children (1880), p. 496, recommends equally useless doses of arsenic. "A child of eight years can take 5 drops, diluted in water, three times daily, after eating; and the dose may be increased, if needed, to eight drops."

Hammond, Diseases of the Nervous System (1881), p. 745, correctly describes the arsenical treatment of chorea by giving Fowler's solution *per os*, and by hypodermic injection.

The internal doses he increases by 1 drop every three days, which in my opinion is too slowly. He gives his preference to the hypodermic injections of diluted Fowler's solution in doses varying from 10 to 30 minims. (gm. 0.6 to 1.8.)

Personal experience:

My own experience is in substantial accord with that of Radcliffe and of Begbie, in that I have almost never known arsenic to fail to cure chorea, and often very quickly. I have almost always given Fowler's solution by the stomach in doses ranging from 3 to 30 drops three times a day.

It is exhibited largely diluted with water, usually half a tumblerful or from 3 to 4 ounces (gm. 90 to 120), and given after food, although I am now inclined to think that the importance of this latter caution has been over-estimated, and is not as great as is that of proper dilution.

In children who are delicate and in sensitive adults I sometimes commence with a dose of 2 drops after each meal; more usually, however, with a dose of 5 drops. Each day I add 1 drop; thus: On the first day the patient takes 5 drops three times a day, on the second day 6 drops three times a day, the third day 7 drops three times, and so on.

Usually, when a dose of from 10 to 14 drops three times a day is attained, some arsenical symptoms appear; these are diarrhœa, nausea, vomiting, anorexia, redness and puffiness about the eyes; one of these symptoms or any combination of them. They are not serious, and during their prevalence I have never found albumen in the urine. My practice formerly was to go back to smaller doses when this condition developed and to again increase; but in the last two years I find it more advantageous to withhold all arsenic for forty-eight hours and then resume at the last dose and begin a further increase. A remarkable tolerance is now shown by most patients, even by young children; and doses of 20, 25, and even 30 drops thrice a day may be reached without a renewal of these symptoms.

I recently had a chronic and relapsing case of hemi-chorea in a girl fourteen years old which resisted doses of 34 drops three times a day, an equivalent of one grain (gm. 0.06) of arsenious acid per diem. My directions as to physical and mental rest were disregarded by parent and patient, and this may account for the failure of the arsenic. Under the use of 2 grains (gm. 0.13) and 3 grains (gm. 0.19) doses of bromide of zinc the movements ceased within a month after the discontinuance of the arsenic.

I have been taught by experience not to expect amelioration of the choreic movements until the toxic effects of arsenic are evident; and in old or relapsing cases, not until the second period of toxic symptoms. I nearly always combine rest, nearly absolute rest in bed, in severe cases, with the arsenical treatment, and, if the patient be wakeful or nervous at night, an occasional bed-time dose of chloral hydrate *per os* or by enema is given.

Simple, acute chorea may often be cured in two weeks' time by this plan, in positive contradiction to the too prevalent notions of self-limitation of chorea and skepticism as to the efficacy of drugs in this disease.

As regards possible ill-effects from this arsenical treatment, I would repeat that, though I have examined the urine of many patients at the period of saturation by arsenic, I have never found albumen or tube-casts. Stomatitis I have seen but once, in the case of a physician's son, whose chorea was very rapidly and permanently cured by moderate doses (about 18 drops three times a day). Cutaneous symptoms I have never met with; and the gastro-intestinal irritation has never been serious or permanent.

My experience with the hypodermic injection of Fowler's solution is more limited. I have made use of it in chronic chorea, and in local choreiform affection, and I would agree with Dr.

Hammond in his statement that we relatively obtain less constitutional disturbance and more curative effect from this method. —*Transactions of the Medical Society of the State of New York for* 1882.

